# Rheology and tribology of starch + *κ*‐carrageenan mixtures

**DOI:** 10.1111/jtxs.12570

**Published:** 2020-11-21

**Authors:** Kwan‐Mo You, Brent S. Murray, Anwesha Sarkar

**Affiliations:** ^1^ Food Colloids and Bioprocessing Group, School of Food Science and Nutrition University of Leeds Leeds UK

**Keywords:** biopolymer mixtures, corn starch, polysaccharides, rheology, tribology, *κ*‐carrageenan

## Abstract

In this study, we investigated the rheological and tribological properties of biopolymer mixtures of gelatinized corn starches (0.5 – 10.0 wt%) and *κ*‐carrageenan (*κ*C) (0.05 – 1.0 wt%). Two different starch samples were used. The first starch (CS1), despite extensive heating and shearing contained “ghost” granules, while the second starch (CS2) had no visible ghost granules after the same gelatinization process as CS1. Apparent viscosity measurements demonstrated that *κ*C + CS1 mixtures were shear thinning liquids, with viscosity values being lower than the corresponding weight average of the values of the individual equilibrium phases at shear rates < 50 s^−1^. Tribological results revealed that *κ*C ≥ 0.5 wt% was required to observe any decrease in friction coefficients in the mixed lubrication regime. Starch (CS1) showed an unusual behavior at ≥ 5 wt%, where the friction coefficient decreased not only in the mixed regime but also in the boundary regime, probably due to the presence of the “ghost” granules, as the latter became entrained in the contact region. The CS1 + *κ*C mixtures showed significantly lower friction coefficients than that of pure CS1 and *κ*C in the mixed regime. However, the CS2 + *κ*C mixture (i.e., containing no ghost granules) showed similar behavior to pure *κ*C in the mixed regime, while lower friction coefficients than that of the pure CS2 and *κ*C in the boundary regime. These findings illustrate new opportunities for designing biopolymer mixtures with tunable lubrication performance, via optimizing the concentrations of the individual biopolymers and the gelatinization state of the starch.

## 1 INTRODUCTION

Tribology is the science of friction, wear, and lubrication of surfaces in relative motion (Ludema & Ajayi, 2018). Such surface interactions can control the function of practically every macroscopic systems with interacting moving parts and are of the utmost importance to various engineering applications, such as gears, bearings, seals, clutches, couplings, and cams (Hutchings & Shipway, 2017; Mang, Bobzin, & Bartels, 2011). In the food community, the importance of “interacting surfaces in relative motion” has been increasingly recognized with respect to oral processing, that is, the processing of food in the mouth, such as tooth‐tooth, tongue‐palate, tongue‐tooth, tooth‐food, tongue‐food, lip‐lip, lip‐food interactions, and so on. More importantly, many of these surface interactions often tend to explain mouthfeel attributes, such as smoothness, pastiness, astringency, which are not solely driven by the bulk rheological properties of the food and cannot be fully explained by measuring apparent viscosity (Laguna & Sarkar, 2017; Pradal & Stokes, 2016; Prakash, Tan, & Chen, 2013; Sarkar & Krop, 2019).

Oral processing includes both rheological and tribological phenomena (Sarkar, Andablo‐Reyes, Bryant, Dowson, & Neville, 2019). In the early stages of oral processing, bulk rheological phenomena could be dominant where the food is considered to be in the continuum, which is measured at a fixed gap, equivalent to the size of the food material. However, with time, oral processing involves a range of deformation process, and the properties of food are not only driven by the viscosity, but also the friction between the surfaces of the oral tissues and the food as well as between the surfaces of the tissues (Sarkar et al., 2019; Stokes, Boehm, & Baier, 2013). Therefore, the tribological properties become dominant in the later stages of oral processing involving surface interactions of the food driving the mouthfeel perception. Therefore, oral tribology has become an increasingly important part of oral processing studies of model and real foods (Laguna, Farrell, Bryant, Morina, & Sarkar, 2017; Sarkar, Andablo‐Reyes, Bryant, Dowson, & Neville, ; Stokes et al., 2013; Stribiţcaia, Krop, Lewin, Holmes, & Sarkar, 2020).

There has also been a gradual increase in interest in understanding specifically the tribological properties of polysaccharides, since they can play an essential role in adjusting the mouthfeel of foods and beverages (Garrec & Norton, 2012, 2013; Stokes, Macakova, Chojnicka‐Paszun, De Kruif, & De Jongh, 2011; Torres et al., 2019; Zhang et al., 2017). Starch is the most widespread polysaccharide in foods that affects food structure and texture and starch textural properties tend to vary widely depending on their origin, for example, corn (maize), wheat, potato, tapioca, and rice. In addition, the textural properties of starches of one origin depend on the starch granule size, shape, degree of swelling, disruption, that is, gelatinization. (Blazek & Gilbert, 2011; Buléon, Colonna, Planchot, & Ball, 1998). The degree of gelatinization may determine the tribological properties, for example, stickness, slipperiness, and so forth (Ai & Jane, 2015; Evans & Lips, 1992), though there have been relatively few systematic studies on the lubrication properties of starch (Torres, Andablo‐Reyes, Murray, & Sarkar, 2018; Torres, Tena, Murray, & Sarkar, 2017; Zhang et al., 2017).

Zhang et al. (2017) studied the tribological properties of suspensions of cooked swollen starch granules (ghost suspensions) from maize or potato across a wide range of concentration. The coefficient of friction (*μ*) was shown to decrease in the boundary and mixed regimes with increasing concentration (0.01 – 1.0 wt%) because the ghost particles became entrained in the contact zone at low entrainment speed (*U*, 40 mm s^‐1^) as compared to that of water, where the latter was squeezed out of the hydrophobic tribological surfaces used in their study. Torres et al. (2018), on the other hand, investigated the tribological effects of wheat starch‐based microgel particles. The magnitude of *μ* decreased on increasing starch content of the microgels from 15 to 20 wt%, the latter particles having been formed from a bulk gel with a higher shear modulus. It was postulated that these higher starch content microgels might therefore be slightly stiffer and better at keeping the hydrophobic tribological surfaces apart, enabling lowering the *μ* values.

The lubricating behavior of non‐starch polysaccharides, such as locust bean gum, carrageenan, gellan, guar, pectin, xanthan gums, and so forth in the boundary and mixed lubrication regimes has also attracted recent research attention. In particular, the tribological properties of *κ*‐carrageenan (*κ*C) have been investigated (Garrec & Norton, 2013; Stokes et al., 2011). Garrec, Guthrie, and Norton (2013) showed that as the concentration of *κ*C increased, the polymer was entrained in the contact zone, and *μ* values decreased at the site of the converging geometry formed between the tribopairs. Interestingly, the lubrication behavior with *κ*C was consistent across smooth to rough PDMS substrates (mean asperity radius 10–400 nm), highlighting their ability to entrain and prevent direct PDMS–PDMS asperity contact, irrespective of the degree of surface roughness (Stokes et al., 2011).

Although some information on the tribological properties of biopolymer solutions is available in the literature, studies of combinations of biopolymers are very rare. On the other hand, there is extensive literature on the phase separation of biopolymer mixtures. Biopolymers phase separate due to thermodynamic incompatibility (Capron, Costeux, & Djabourov, 2001; Firoozmand, Murray, & Dickinson, 2007; Lorén et al., 2001; Murray & Phisarnchananan, 2016; Semenova & Dickinson, 2010; Vis et al., 2015), giving rise to two‐phase systems. Previous studies have shown that such phase separation leads to modification of the bulk rheology of binary mixtures, related to excluded volume effects between the two biopolymers, such as starch and *κ*C, which mutually exclude each other in solution (Fakharian, Tamimi, Abbaspour, Nafchi, & Karim, 2015; Huc et al., 2014; Lafargue, Lourdin, & Doublier, 2007; Tecante & Doublier, 1999). Thus, both the storage modulus (*G*^′^) and loss modulus (*G*^"^) can be much higher in mixtures than with *κ*C alone (Fakharian et al., 2015; Lafargue et al., 2007). Also, a slight increase in apparent viscosity of the mixtures compared to *κ*C alone has been observed (Huc et al., 2014). Thermodynamic incompatibility between starch and other polysaccharides has been extensively described previously (Chen et al., 2018; Firoozmand et al., 2007; Murray & Phisarnchananan, 2016; Tecante & Doublier, 2002).

In this study, we used corn starch (CS) and *κ*C as model biopolymers to investigate the rheological and tribological properties of the biopolymer mixture. We measured the shear viscosity, load‐bearing properties and Stribeck curves of *κ*C and CS individually and then the properties of mixtures of the two. In particular, two types of CSs were employed, one containing ghost granules and another containing no ghost granules post gelatinization, to understand the effect (if any) of these intact starch granules on the tribological properties of the biopolymer mixtures. Ghost granules, or ghost remnants, are derived from the external layers of the granules of various starches, depending on the ratio of amylose /amylopectin present in the starch. They are shown to exhibit elastic/plastic properties, therefore, they are likely to affect oral processing and even lubrication (Atkin, Abeysekera, & Robards, 1998).

To our knowledge, this is the first study that reports the frictional properties of biopolymer mixtures of CS and *κ*C and the findings bring new knowledge for the design of biopolymer mixtures with tailored lubrication performance.

## 2 MATERIAL AND METHODS

### 2.1 Materials

*κ*‐carrageenan (*κ*C), product code 22048 (CAS number 11114‐20‐8), corn starch (CS1), product code S9679 (CAS number 9005‐25‐8) and the second corn starch sample (CS2) 10120 (CAS number 9037‐22‐3), both derived from maize, were all purchased from Sigma‐Aldrich, Dorset, United Kingdom. Although both CS1 and CS2 contain mostly amylopectin, CS1 contained residual ghost granules after heating which was not there in CS2. Polysaccharide solutions were prepared in 20 mM phosphate buffer at pH 7.0. Smooth polydimethylsiloxane (PDMS, Sylgard 184, Dow Corning, United States) tribo‐couples, that is, ball (∅ 47 mm) and disc (∅ 19 mm, 4 mm thickness), with the surface roughness of 50 nm, were purchased from PCS Instruments, London, United Kingdom. Rhodamine B (product code R‐6626) was purchased from Sigma Aldrich. Water purified by a Milli‐Q apparatus (Millipore, Bedford, United Kingdom), with a resistivity not less than 18.2 MΩ.cm, was used for the preparation of the buffer and any other solutions.

### 2.2 Preparation of starch + *κ*C mixtures

Gelatinized waxy corn starch (CS1 or CS2) (0.5 – 5 wt%) was prepared by dispersing the starch powder in phosphate buffer at pH 7.0, followed by heating in a water bath at 90°C for 20 min with constant shearing using a magnetic stirrer, to gelatinize the starch*. κ*‐carrageenan (*κ*C) was similarly dispersed in phosphate buffer at pH 7.0 (0.05 – 1.0 wt%) for at least 24 h at room temperature and then heated at 90°C for 20 min. To prepare the biopolymer mixtures, both solutions were prepared separately as above before mixing. Equal volumes of starch solution and *κ*C solution of different concentrations were blended and homogenized at 21,000 rpm for 30 min using an Ultra Turrax T25 homogenizer (IKA‐Werke GmbH &Co., Staufen, Germany) at room temperature (25°C).

### 2.3 Apparent viscosity

Rheological characterization of the pure biopolymers and their mixtures was performed using a modular compact rheometer, model MCR 302 (Anton Paar, Austria) at shear rates ranging from 0.1 to 1,000 s^−1^ at 37°C. The biopolymer mixtures did not phase separate within the first 2 h of preparation but started to separate after 7 days of storage at ambient conditions (see Figure S1) and all the rheological experiments were carried out within 2 h of preparation of the biopolymer mixtures. A cone‐and‐plate geometry system (CP50‐2, cone diameter 50 mm, cone angle: 2°) with a gap of 1 mm was used for all measurements. For each measurement, 2 ml of sample were pipetted onto the plate, excluding any air bubbles. A temperature‐controlled cover prevented evaporation during the measurements and helped to maintain the temperature at 37°C (i.e., representing oral processing temperature). Samples were left on the plate for ~2 min to achieve a steady state, following which the apparent viscosities were measured. High shear rate limiting viscosity (*η*_∞_) was determined to scale the tribological data in the Stribeck curves (see later in Section 3).

### 2.4 Tribology

Tribological measurements on the pure biopolymers and their mixtures samples were performed using a Mini Traction Machine 2 (MTM2, PCS Instruments) with hydrophobic polydimethylsiloxane (PDMS) ball and disc as tribopairs. All the tribological experiments were carried out within 2 h of preparation of the biopolymer mixtures where the mixtures were in a single phase (Figure S1). A normal load (*W*) of 2 N and a slide‐to‐roll ratio (SRR) of 50% were set for all Stribeck measurements. The sliding speeds were varied from 1 to 0.001 m s^‐1^. The coefficient of friction was measured for all samples as a function of entrainment speed. The entrainment speed *U* is defined as in Equation (1):

(1) $$\;U=\frac{1}{2}\;\left(U_{B}+U_{D}\right)$$

where, *U*_B_ is the rolling speed of the ball and *U*_D_ is the sliding speed of the disc. The temperature in the tribological experiment was set at 37 ± 1°C, matching that of the rheological measurements. In addition, the load‐bearing ability of the biopolymers was also measured at loads (*W*) ranging from 1 to 5 N, at *U* = 0.005 m s^‐1^. The friction coefficients are reported as the mean and *SD* of 3 measurements carried out on at least triplicate samples prepared on different days.

### 2.5 Microscopy

Optical microscopy (Nikon, SMZ‐2 T, Japan) was used to observe the microstructure of the heated starch samples to identify any residual granule structures. Samples were diluted with buffer (1:10 wt/wt). A confocal laser scanning microscope (Model LSM 880, Carl Zeiss MicroImaging GmbH, Jena, Germany) was also used for microstructural characterization of some samples, after mixing with 0.5 wt% Rhodamine Blue (RB), excited at 514 nm, to fluorescently label the starch. Samples were excited with a He/Ne (543, 633 nm) laser source. A 20× objective with numerical aperture 0.5 was used to obtain all images, at 1,024 × 1,024 pixel resolution.

### 2.6 Statistics

All experimental results were reported as mean and *SD*s of three measurements on triplicate samples (*n* = 3 × 3). The statistical analyses were conducted for the rheological data at 50 s^−1^ simulating oral processing shear and tribological data at boundary regime (0.005 m/s) and mixed regimes (0.05, 0.1 m s^‐1^) using one‐way analysis of variance and multiple comparison test using SPSS software (IBM, SPSS statistics, version 24) and the significant difference between samples were considered when *p* < .05 using Tukey's test. Statistical results can be observed in Tables S1a–d and S2a–c.

## 3 RESULTS AND DISCUSSION

### 3.1 Tribological and rheological properties of pure biopolymers

**FIGURE 1:**
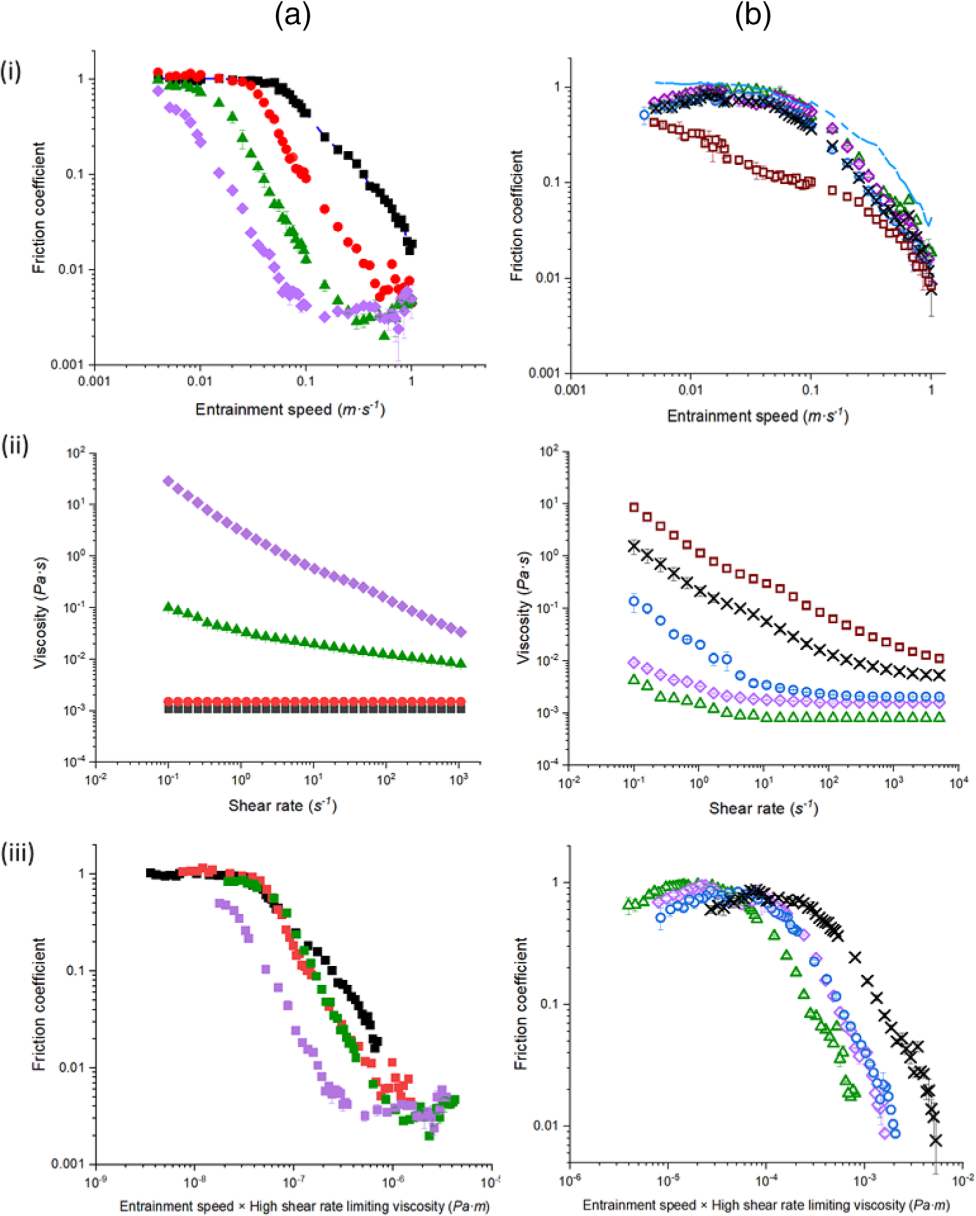
(i) Friction coefficient (*μ*) versus entrainment speed (*U*), (ii) apparent viscosity (*η*) versus shear rate (*γ*), and (iii) friction coefficient (*μ*) as a function of product of entrainment speed and effective viscosity (*Uη*) of (a) *κ*C and (b) CS1 at various concentrations (*κ*C: 0.05 wt% (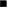 ), 0.1 wt% (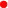 ), 0.5 wt% (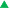 ), and 1.0 wt% (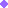 ); CS1: 0.5 wt% (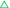 ), 1.0 wt% (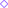 ), 2.0 wt% (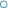 ), 3.0 wt% (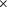 ) and 5.0 wt% (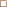 ). Phosphate buffer is used as a control (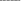 ). Values represent means and error bars represent the *SD*s for at least three measurements on triplicate samples (*n* = 3 × 3)

Figure 1ai and bi illustrate the friction coefficients (*μ*) as a function of entrainment speed (*U*) of *κ*C (0.05 – 1.0 wt%) and CS1 (0.5 – 5.0 wt%), respectively. The phosphate buffer on its own (Figure 1ai) shows a prolonged boundary regime with *μ* ≈ 1.0 until *U* ≈ 0.1 m s^‐1^ followed by a decrease in *μ* as the mixed lubrication regime commences, where the pressure in the contact region between the PDMS tribopairs is sustained partly by the fluid and partly by the surfaces. Such a prolonged boundary regime with phosphate buffer has been seen previously (Sarkar, Kanti, Gulotta, Murray, & Zhang, 2017), where phosphate buffer was proposed to be squeezed out of the hydrophobic PDMS–PDMS contact zone.

The *κ*C solutions (Figure 1ai) showed interesting concentration‐dependent tribological properties, where the extents of the boundary regime were shortened, that is, the onset of mixed regime occurred at lower speeds, upon increasing the concentration of *κC* from 0.05 to 1.0 wt% with no changes in boundary friction observed from 0.05 to 0.5 wt% concentration (*p* > .05) (see Table S1a for statistical analyses). Also, the *μ* values in the mixed lubrication regime decreased sharply as the concentration was increased from 0.05 wt% to 0.1 wt% (*p* < .05) but no significant decline was observed when the concentration increased from 0.5 wt% to 1.0 wt% (*p* > .05) (see Table S1a for statistical analyses). For instance, the *μ* values decreased by an order of magnitude at *U* = 0.1 m s^‐1^, when the concentration of *κ*C was increased by order of magnitude, that is, from 0.1 to 1.0 wt%. Stokes et al. (2011) have already indicated that *κ*C is a useful potential lubricant because it decreases friction coefficients efficiently in the mixed regime, compared to numerous other aqueous polysaccharides (pectins, locust bean gums). It is noteworthy that *κ*C solutions ≥ 0.5 wt% also demonstrated a characteristic hydrodynamic lubrication regime behavior, where the *μ* values started to increase at the highest *U*.

Shifting our focus to starch, 0.5 – 2.0 wt% CS1 (Figure 1bi) showed similar boundary behavior to that of the phosphate buffer (up to *U* = 0.1 m/s), but slightly lower *μ* values in the mixed lubrication regime as compared to that of the buffer. Interestingly, *μ* was more or less the same for all CS1 concentrations between 0.5 and 2.0 wt% irrespective of the regimes (*p* > .05) (see Table S1b for statistical analyses), unlike the behavior with *κ*C (Figure 1ai), the latter showing a strong concentration‐dependence on frictional behavior. However, *μ* values at 5.0 wt% CS1 (Figure 1bi) were significantly lower than for the other starch concentrations in the mixed regime at *U* = 1 m s^‐1^ (*p* > .05) (see Table S1b for statistical analyses), but converged on the same values as *U* approached the maximum applied (*U* = 1 m s^‐1^). Particularly CS1 at 5.0 wt% did not show any visible boundary regime. This behavior might be due to the presence of higher concentrations of “ghost” granules at the higher CS1 concentration, which potentially might have granule‐granule inter‐molecular friction, flattened and providing sufficient hydrodynamic lift to separate the PDMS surfaces leading to the immediate onset of the mixed lubrication regime even at low speeds (*U* ≤ 0.005 m s^‐1^).

**FIGURE 2:**
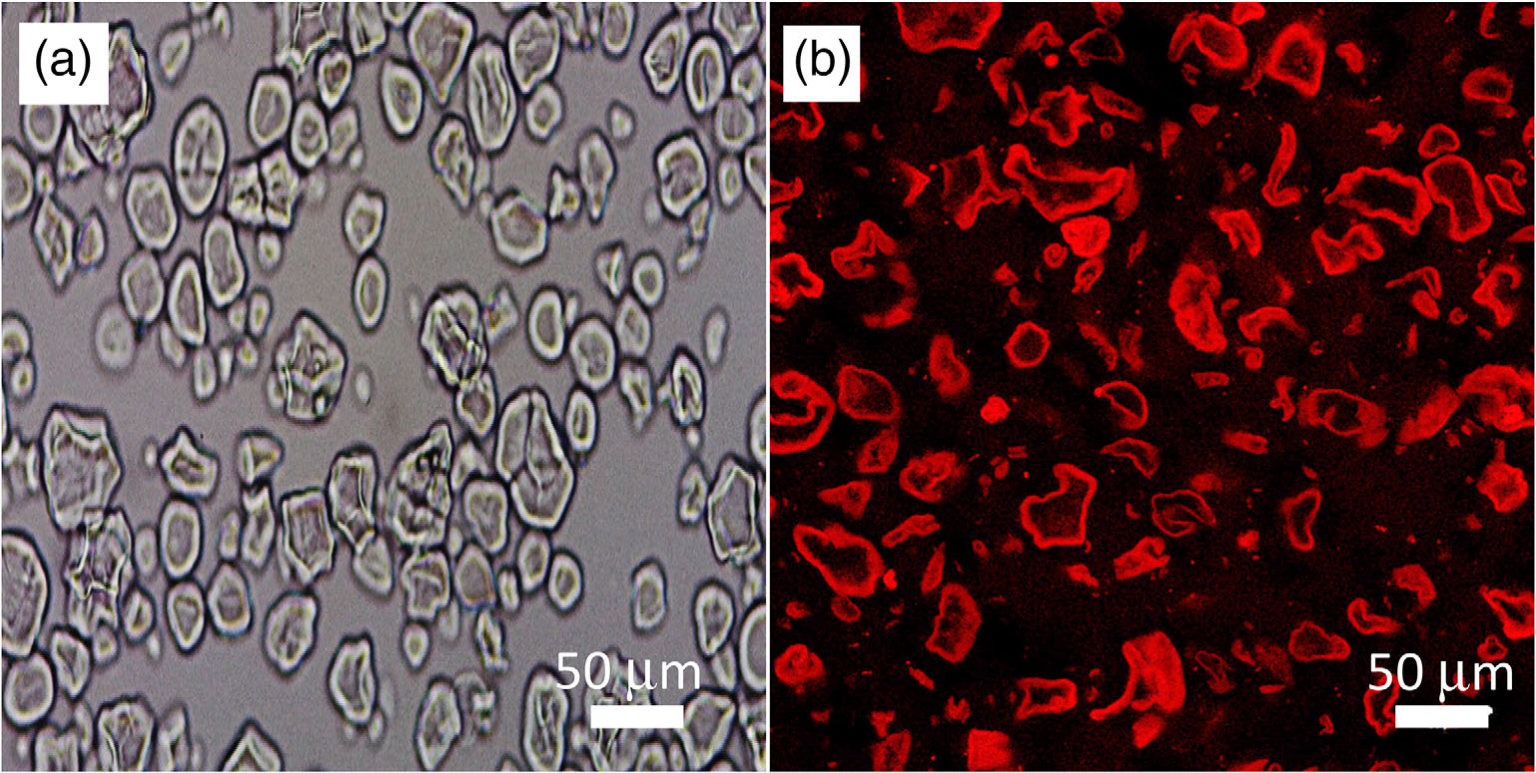
Optical (a) and confocal (b) micrographs of 1 wt% CS1 after gelatinization. The bright regions in (b) are due to CS1 labeled with Rhodamine Blue. Scale bar is 50 μm

The presence of ghost granules in the CS1 sample was evidenced via optical and confocal laser scanning microscopy, as shown in Figure 2a,b, respectively. It is clear from both these images that ghost granules remained in the CS1 sample even though the starch was subjected to high temperature (≈ 90°C) and shearing.

Liu, Stieger, Van Der Linden, and Van De Velde (2016) have reported that the tribological properties of rice starch in liquid and semi‐solid model food systems are often associated with the soft and deformable nature of the gelatinized starch granules—that can flatten and fill the asperities between the PDMS–PDMS contact region in the boundary regime. This creates a smoother surface contact zone. In addition, the gelatinized rice starch granules in the afore‐mentioned study formed a thicker continuous tribological film that separated the tribo‐pair surfaces due to the increased viscosity. Therefore, the deformability of ghost granules, which are tens of microns in size, plus their potential ability to form a more continuous film, could explain the absence of the boundary regime and rapid onset of mixed lubrication regime seen (Figure 1bi) for 5.0 wt% CS1.

It is also essential to check the lubricant's bulk viscosity, which is crucial in determining the film thickness. The viscosity of the fluid, particularly at high shear rates, is one of the key factors that affect film thickness, which in turn affects the friction coefficients of biopolymers in the mixed and elastohydrodynamic regimes, as highlighted by Stokes et al. (2011) and Andablo‐Reyes et al. (2019). If the viscosity of the biopolymer is too low, the resulting film thickness will not be sufficient to provide adequate separation between the two surfaces. To address this, Figure 1aii,bii show the flow curves of different concentrations of *κ*C and CS1 solutions, respectively. As one might expect, higher viscosity values occurred at higher concentrations of the biopolymers (see Tables S2a,b for statistical analyses) and both *κ*C and CS1 showed shear thinning behavior. However, marked shear thinning only occurred for CS1 at higher concentrations (≥ 3.0 wt%) and the viscosity was significantly higher than those of 0.5 – 2.5 wt% CS1 at the typical oral processing shear rate of 50 s^−1^ (see Table S2b for statistical analyses). In order to estimate the viscous contribution in the tribological data, the high shear rate limiting viscosity (*η*_∞_) was obtained. The Reynolds equation for soft‐elastohydrodynamic lubrication (EHL) was applied when the sample showed a clear hydrodynamic regime (iso‐viscous‐elastic lubrication regime), as observed for *κ*C in Figure 1ai at ≥ 0.5 wt%. This model (De Vicente, Stokes, & Spikes, 2005) gives an arithmetic expression of the Reynold's equation for the soft EHL lubrication regime between sliding‐rolling ball and plate contact under fully flooded conditions. The friction coefficient in contact can be expressed in terms of the characteristic parameters: *W*, *η* and *U*, as follows (Equation 2):

(2) $$\mu =1.46\;\frac{{\left(\mathrm{\eta U}\right)}^{0.65}W^{-0.76}}{{E^{*}}^{1.35}{R^{*}}^{2.05}}+\mathrm{SRR}\left(3.8\frac{{\left(\mathrm{\eta U}\right)}^{0.71}W^{-0.76}}{{E^{*}}^{1.47}{R^{*}}^{2.23}}+0.96\frac{{\left(\mathrm{\eta U}\right)}^{0.36}W^{-0.11}}{{E^{*}}^{0.47}{R^{*}}^{0.58}}\right)$$

where $E^{*}={\left(\frac{1-{v_{1}}^{2}\;}{E^{\prime }}+\frac{1-{v_{2}}^{2}\;}{E^{\prime \prime }}\right)}^{-1}$ and $R^{*}={\left(\frac{1}{R^{\prime }}+\frac{1}{R^{\prime \prime }}\right)}^{-1}$ are the reduced Young's modulus and reduced radius of the contact, respectively. Here *E*^′^ and *E*^′′^ are the elastic moduli of the surface material and *R*^′^ and *R*^′′^are the radius of the surfaces.

The first term in Equation (2) is the Poiseuille contribution to the friction, while the second corresponds to the Couette friction. Stokes et al. (2011); Andablo‐Reyes et al. (2019) showed that Equation (2) can be successfully fitted to the experimental data in Figure 1aii, and consequently, the value of *η*_∞_ obtained. In our study, *SRR* = 0.5, *E*^′^ of the PDMS ball and disk ≈ 2.4 MPa and *R*^′^ (= *R*^′′^) = 9.5 × 10^−3^ m. The calculated values of *η* for 0.5 and 1.0 wt% *κC* were 0.00267, and 0.004 Pa s, respectively. Using these estimated values of *η* (*η*_∞_), we plotted the corresponding Stribeck curves (Figure 1aiii) to identify the concentrations of *κ*C where the tribology was dominated by the bulk rheological properties. Figure 1aiii shows that 0.1 – 0.5 wt% *κ*C gave similar shaped curves with overlapping characteristics, which means that the tribological properties of these solutions were dominated by their *η*_∞_ (Figure 1aiii). However, at concentrations ≥0.5 wt% *κ*C, there was a lower *μ* in the mixed lubrication regime, which might be attributed to the coil‐helix transition of *κ*C as it transforms from a solution to a gel (Gabriele, Spyropoulos, & Norton, 2009; Garrec & Norton, 2013; Rochas & Landry, 1987; Rochas & Rinaudo, 1982), allowing *κ*C to entrain into the contact zone fully.

For CS1 (Figure 1biii), the Stribeck curve obtained using Equation (2) did not show good agreement with increasing concentration of CS1, the Stribeck curves being significantly shifted to the right of the data points. These results support the idea that the stickiness of starch granules in the dispersions might resist the relative motion of the tribo‐pairs due to molecular adhesion as reported by previous studies (Liu et al., 2016; Wu, Gunaratne, Collado, Corke, & Lucas, 2015; Zhang et al., 2017), although this needs further investigation.

### 3.2 Load‐bearing abilities of the biopolymers

**FIGURE 3:**
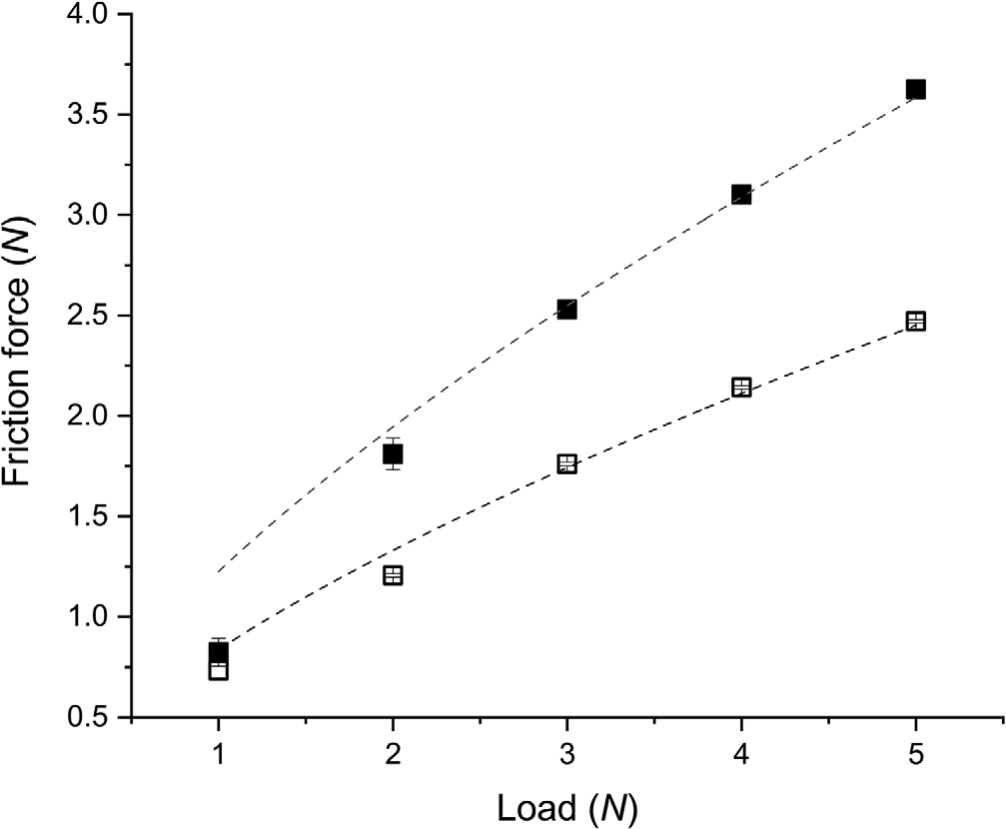
Friction force versus applied load for *κ*C (■) and CS1 (□) when sheared between polydimethylsiloxane (PDMS) ball and disc at a constant speed of 0.005 m/s. Values represent means and error bars represent the *SD*s for at least three measurements on triplicate samples (*n* = 3 × 3)

The load‐bearing capacity of the lubricating film is an important parameter to help understand the lubrication performance of biopolymers. The friction force (*F*) of *κ*C and CS1 as a function of *W* at low *U* (0.005 m s^‐1^) is shown in Figure 3, aiming to test the load‐bearing abilities of the biopolymers (see *μ* values of *κ*C and CS1 as a function of *U* at different *W* in Figure S2). According to Stokes et al. (2011), *μ* for an adsorbed polysaccharide scales with *W* as *μ* ~ $W^{\frac{2}{3}}$. This dependency originates from Equation (3):

(3) $$F=\tau_{i}\times A$$

where *A* = the contact area for a circular contact $=\pi {\left(\frac{3\;WR^{*}}{4E^{*}}\right)}^{\frac{2}{3}}$; *τ*_*i*_ is the interfacial shear stress between the surfaces. The *F* versus *W* data in Figure 3 were thus fitted using Equation (4):

(4) $$F=8.5\cdot 10^{-6}\cdot \tau_{i}\times W^{\frac{2}{3}}$$

The dashed lines in Figure 3 show the best fits and the slope of these lines gives *τ*_*i*_, which for *κ*C and CS1 were 0.144 and 0.099 MPa, respectively, that is, lower than the value for PDMS/water/PDMS (*τ*_*i*_ = 0.23 MPa). Thus CS1 lowers the interfacial shear stress between the surfaces even more than *κ*C suggesting that CS1 is more likely to remain bound to the PDMS surfaces. This further explains the larger decrease in friction coefficient with CS1 and the almost non‐existent boundary lubrication regime illustrated in Figure 1bi.

### 3.3 Rheological versus tribological behavior of starch + κC mixtures

**FIGURE 4:**
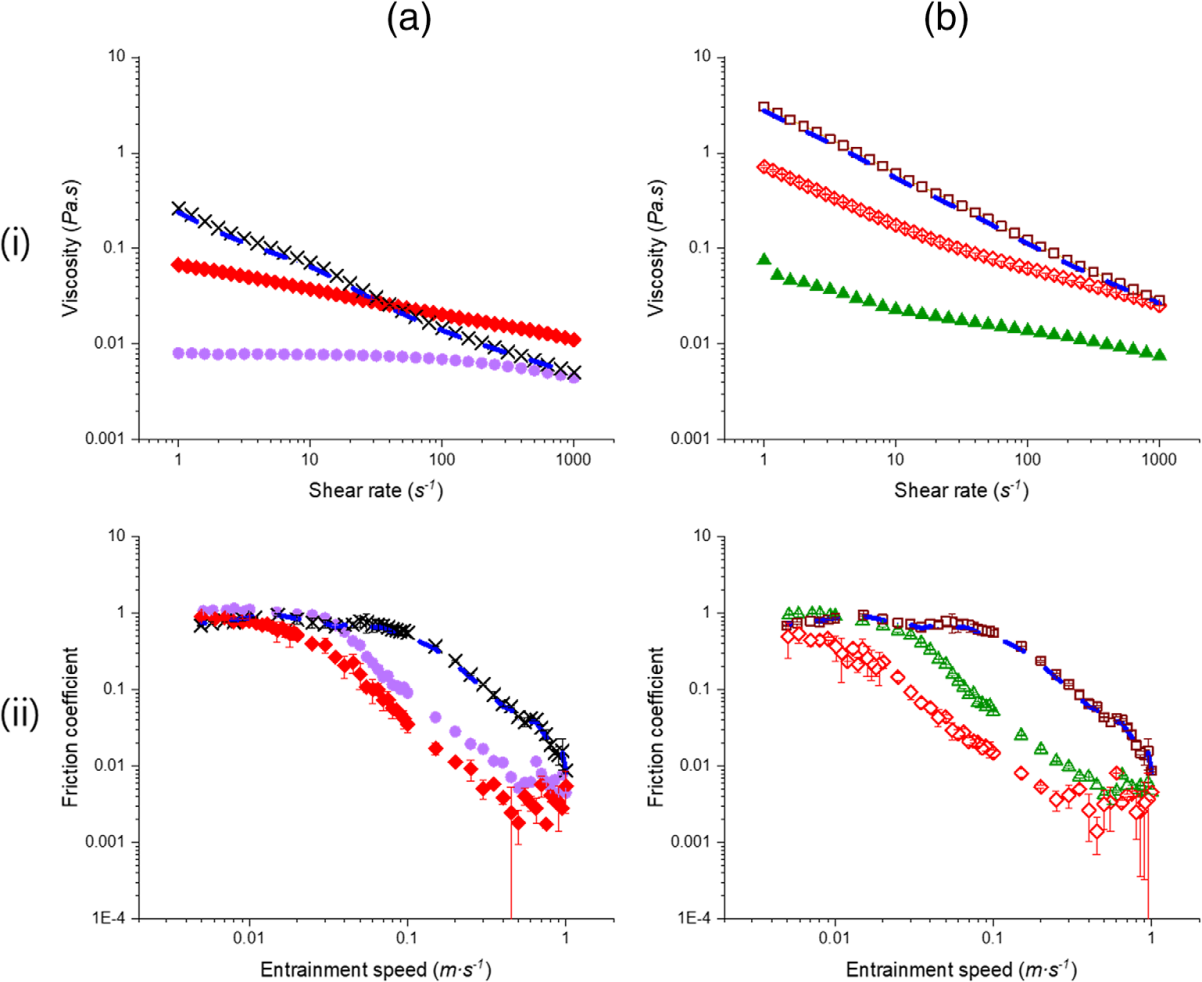
(i) Apparent viscosity (*η*) as a function of shear rate (*γ*) and (ii) friction coefficients (*μ*) versus entrainment speed (*U*) of biopolymer mixtures at (a) lower biopolymer concentrations: (1.5 wt% CS1 + 0.15 wt% *κ*C, 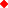 ) and (b) high biopolymer concentrations (2.5 wt% CS1 + 0.25 wt% *κ*C, 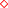 ) plus the controls of 0.15 wt% *κC* (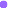 ), 0.25 wt% *κC* (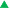 ), 1.5 wt% CS1 (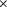 ) and 2.5 wt% CS1 (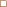 ) alone. The weight average values of the corresponding individual controls for the mixtures are also shown (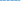 ). Values represent means and error bars represent the *SD*s for at least three measurements on triplicate samples (*n* = 3 × 3)

Both Figure 4ai,bi demonstrate that the CS1 + *κ*C mixtures, irrespective of the total biopolymer concentration (1.65 or 2.75 wt%) have non‐Newtonian behavior and the viscosity values at orally relevant shear rate of 50 s^−1^ were significantly different in comparison to either CS1 or *κ*C (see Table S2c for statistical analyses). As can be seen for both biopolymer concentrations, the viscosity values of the mixture containing the weighted average of the individual equilibrium phases (*κ*C and CS1) were similar to that of the starch alone (see dashed line in both Figure 4ai,bi. Interestingly, the measured viscosity values at either biopolymer concentration (1.65 or 2.75 wt%) were lower than the mixture containing weighted average of the individual equilibrium phases (*κ*C and CS1) at corresponding shear rates, except in case of the 1.65 wt% mixtures at ≥ *ca*. 50 s^−1^ (Figure 4ai). In other words, the viscosity behavior appeared to be inordinately affected by the *κ*C in the mixture. This is in line with the rheological properties of the biopolymer mixtures studied previously, where the viscosity of the mixtures tended to be higher than the value of *κ*C alone (Fakharian et al., 2015; Huc et al., 2014; Lafargue et al., 2007).

Looking at the tribological data, the *μ* of the mixtures (Figure 4aii,bii at 1.65 and 2.75 wt%, respectively) were considerably lower than that of pure CS1 across the whole range of mixed regime (see Table S1c,d for statistical analyses). Most surprisingly, unlike the bulk rheology results (Figure 4ai,bi), the *μ* values of the mixtures were statistically similar to those of pure *κ*C (*p* > .05) except for the 2.75 wt% mixture (*p* < .05) (see Table S1c,d for statistical analyses). Nevertheless, the *μ* values were always lower than the values for the mixtures containing the weighted average of the individual phases (Figure 4aii,bii), irrespective of the total biopolymer concentration. Possibly this is related to local phase separation within the mixtures at the time of measurement, although the measurements were made immediately after mixing and phase separation is normally quite slow to evolve (see Figure S1). No phase separation was visible by eye at the time of measurement but the thermodynamic incompatibility between starch and κC might lead to a locally phase‐separated network in the tribological gap. Overall, how phase separation overall affects the tribological properties of these thermodynamically incompatible biopolymer mixtures remains to be understood. The other possibility is that the behavior is driven by the presence of the starch ghost “granules” observed in the mixture. Tecante and Doublier (1999); Chaudemanche and Budtova (2008) have proposed that non‐penetration between *κ*C and swollen starch granules induces a “excluded volume effect,” in other words swollen starch granules produce an effective increase in the local concentration of *κ*C, which might have caused the reduction in friction irrespective of the total biopolymer concentration (1.65 or 2.75 wt%) (Figure 4aii,bii). In addition, the swollen, more deformable gelatinized granules could entrain in the gap to support the load (Torres et al., 2018).

**FIGURE 5:**
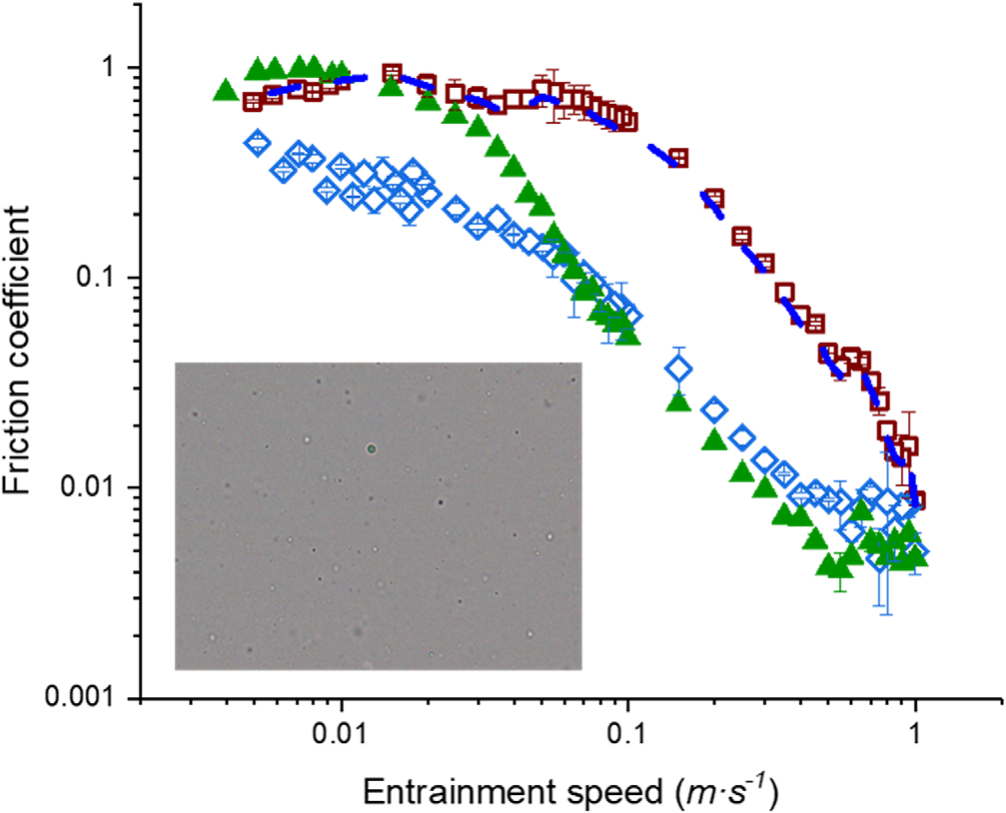
Friction coefficient (*μ*) versus entrainment speed (*U*) of biopolymer mixtures using CS2 without the “ghost” granules, that is, 2.5 wt% CS2 + 0.25 wt% *κ*C, (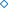 ) and controls of 2.5 wt% CS2 (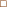 ) and 0.25 wt% *κ*C (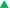 ). Values represent means and error bars represent the *SD*s for at least three measurements on triplicate samples (*n* = 3 × 3). The inset is an optical micrograph of CS2 starch after gelatinization, illustrating the lack of “ghost” granules. The weight average values of the corresponding individual controls for the mixtures are also shown (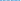 ). Error bars represent *SD*s

To understand further the role of these “ghost” starch granules, Figure 5 shows the *μ* values versus *U* of a mixture of 2.5 wt% CS2 + 0.25 wt% *κ*C, that is, the starch which contained no starch granules. Similar to the mixtures containing CS1 (Figure 4aii,bii), the 2.5 wt% CS2 + 0.25 wt% *κ*C mixture did not show a reduction in friction in comparison to pure *κ*C in the mixed lubrication regime (*p* > .05) (see Table S1d for statistical analyses). Comparing the data of the mixture containing CS2 in Figure 5 with the behavior of CS1 in Figure 4bii, the *μ* values of the CS2 + *κC* mixture appeared to be comparatively higher than those of the CS1+ *κ*C mixture at higher entrainment speeds, though statistically this was not significant (*p* > .05) (see Table S1d for statistical analyses). Nevertheless, it was interesting to observe that the 2.5 wt% CS2 + 0.25 wt% *κ*C mixture (Figure 5) reduced the boundary friction significantly as compared to pure *κ*C and the friction in the mixed regime with respect to CS2 (*p* < .05) (Figure 4bii and Table S1d for statistical analyses).

The inset to Figure 5 is a corresponding optical micrograph of the CS2 starch after gelatinization, clearly illustrating the lack of “ghost” granules, which would therefore appear to be the explanation of this stark difference in behavior compared to CS1. This suggests that the micron‐sized “ghost” granules present in the CS1 + *κ*C mixtures were efficient in reducing the friction in the mixed lubrication regime. However, they were not able to get in between the PDMS surfaces in the boundary region, that is, at low *U*. On the other hand, the CS2 + *κ*C mixture containing no “ghost” granules were beneficial in creating potentially nanometric sized boundary films creating smoother tribo‐contact surfaces. It is worth highlighting that CS2 apparently formed such boundary lubrication films only in presence of *κ*C — such behavior is not seen in pure CS2 (Figure 5). This suggests some local interaction is occurring between CS2 and *κ*C in the boundary region, which requires further investigation in the future.

## 4 CONCLUSIONS

In this study, we investigated the rheological and tribological properties of *κ*‐carrageenan and gelatinized CS and their mixtures. Both *κ*C solutions and gelatinized starch dispersions were shear‐thinning liquids, and one starch (CS1) still contained ghost granules while the other (CS2) did not. In tribological measurements, *κ*C showed good lubrication performance by efficiently reducing the friction coefficient in the mixed lubrication regime, especially when the concentration was ≥ 0.5 wt% and also showed hydrodynamic behavior at higher entrainment speeds (*U*). On the other hand, the CS1 at ≥ 5 wt% immediately showed onset of a mixed lubrication regime at *U* < 0.01 m s^‐1^ without any observed boundary regime. This was attributed to the presence of “ghost” starch granules that flattened in the confinement and enabled accelerated onset of the mixed lubrication regime, which also provided better load‐bearing ability than *κ*C. Mixtures of CS1 + *κ*C (at 1:10 wt/wt ratio) showed that the viscosity values were lower than the weight average of the individual equilibrium phases (CS1 + *κ*C) and the *μ* values of the mixtures containing the ghost starch granules (from CS1) were much lower in the mixed lubrication regime. The mixture CS2 + *κ*C, lacking ghost starch granules, did not offer such lubrication benefits in the mixed lubrication regime. However, CS2 + *κ*C did offer effective boundary lubrication with respect to *κ*C, owing to the gelatinized starch + *κ*C somehow forming a lubricating film. This highlights the crucial nature of the state of gelatinization of the starch in understanding the friction and lubrication properties of such mixtures. Ongoing studies are focusing on identifying the exact mechanisms of the tribological behavior, to allow their fine‐tuning for fat mimetic applications.

## AUTHOR CONTRIBUTIONS

**Kwan‐Mo You:** Data curation; formal analysis; investigation; methodology; validation; visualization; writing‐original draft. **Brent S. Murray:** Methodology; supervision; visualization; writing‐review and editing. **Anwesha Sarkar:** Conceptualization; funding acquisition; methodology; project administration; supervision; visualization; writing‐review and editing.

## ETHICAL STATEMENTS

**Conflict of Interest:** The authors declare that they do not have any conflict of interest.

**Ethical Review:** This study does not involve any human or animal testing.

**Informed Consent:** Not applicable.

## Supporting information


**Appendix** **S1:** Supplementary Information

## Data Availability

The data presented in this article will be openly available from the University of Leeds data repository: https://doi.org/10.5518/927
