# Supplementary material for: Rheology and tribology of starch + κ‐carrageenan mixtures
Source: J Texture Stud. 2020 Nov 21;52(1):16–24. doi: 10.1111/jtxs.12570 (PMC7894308; doi:10.1111/jtxs.12570)
Supplement: Supplementary file 1 — Appendix S1: Supplementary Information [file JTXS-52-16-s001.docx]

**Supplementary Document**

**Rheology and tribology of starch + *κ*-carrageenan mixtures**

*Kwan-Mo You, Brent S. Murray, Anwesha Sarkar**

Food Colloids and Bioprocessing Group, School of Food Science and Nutrition, University of Leeds, Leeds LS2 9JT, United Kingdom

***Corresponding author:**

Prof. Anwesha Sarkar

Food Colloids and Bioprocessing Group,

School of Food Science and Nutrition, University of Leeds, Leeds LS2 9JT, UK

E-mail address: [A.Sarkar@leeds.ac.uk](mailto:A.Sarkar@leeds.ac.uk)

**(a)**

**2 h storage**

**(b)**

**7 days storage**


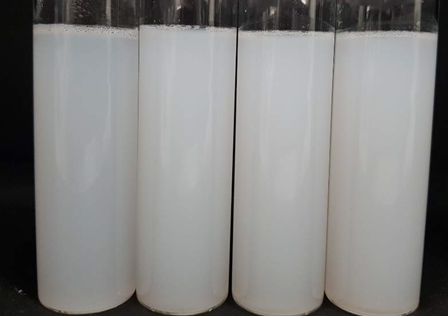

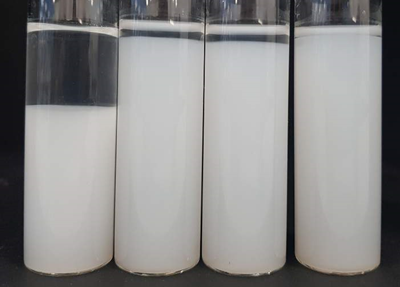


**(i)**

**(ii)**

**(iii)**

**(iv)**

**(iii)**

**(iv)**

**(ii)**

**(i)**

**Supplementary Figure S1.** Visual iamges of 2.5 wt% CS1 + *κ*C mixtures containing (i) 0.025 wt% *κ*C*,* (ii) 0.05 wt% *κ*C, (iii) 0.15 wt% *κ*C, (iv) 0.25 wt% *κ*C after (a) 2 h and (b) 7 days of storage at ambient conditions.

**
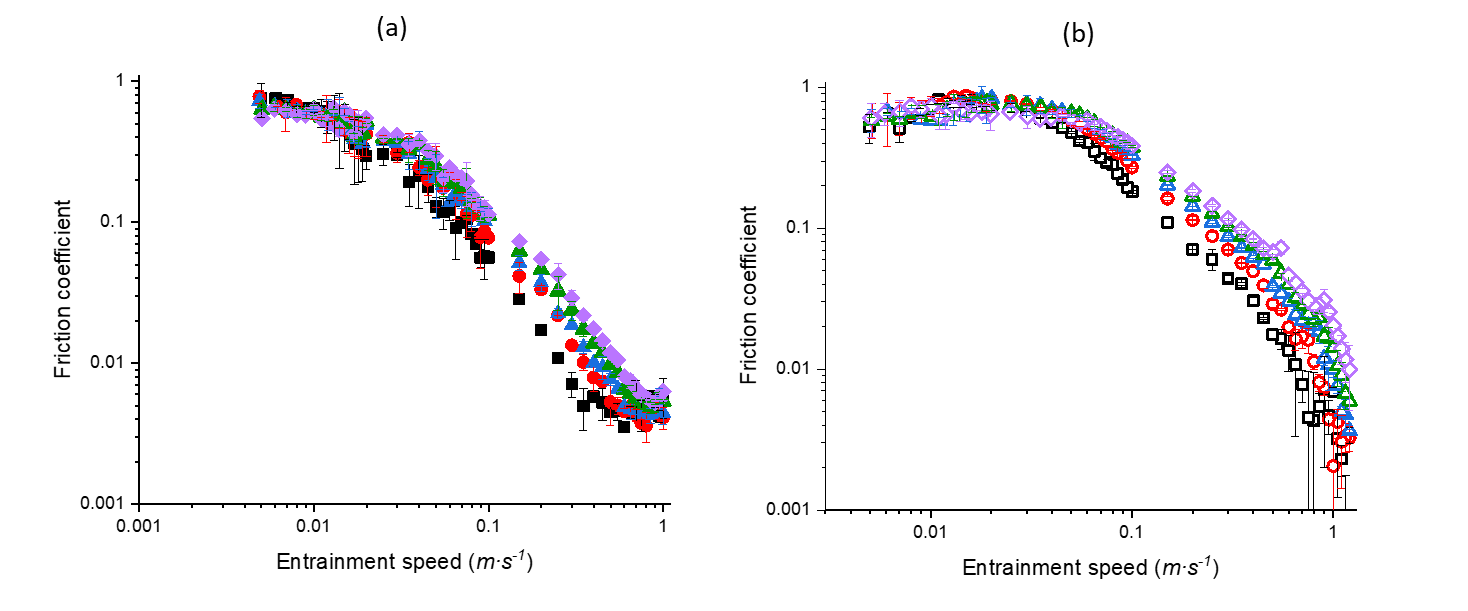
**

**Supplementary Figure S2.** Friction coefficient (*μ*) of (a) 0.5 wt% *κ*C (closed symbols) and (b) 5 wt% G-CS (open symbols) as a function of entrainment speed (*U*) at loads ranging from 1 (■, □), 2 (●, **○**), 3 (▲, **∆**), 4 (▼,**▽**) to 5 (**⯁**,**◇**) N, respectively.

Supplementary Table S1. Mean and standard deviation (SD) of the friction coefficients in the boundary and mixed regimes of *κ*C (a), CS1 (b), the *κ*C + CS1 mixtures with respective concentrations of *κ*C and CS1 (c) and the *κ*C + CS2 mixtures compared with *κ*C + CS1 mixtures with respective concentrations of *κ*C, CS1 and CS2 (d). Different lower case letters in the same column indicate a statistically significant difference (*p < 0.05*).

| **(a)** | **Friction coefficient of *κ*C** | | | | | |
| --- | --- | --- | --- | --- | --- | --- |
|  | Boundary lubrication regime | | Mixed lubrication regime | | Mixed lubrication regime | |
|  | (0.005 m s^-1^) | | (0.05 m s^-1^) | | (0.1 m s^-1^) | |
|  | Mean | SD | Mean | SD | Mean | SD |
| *κ*C 0.05 wt% | 1.0440^a^ | 0.0400 | 0.9320^a^ | 0.0710 | 0.4480^a^ | 0.0400 |
| *κ*C 0.1 wt% | 1.0665^a^ | 0.2000 | 0.3826^b^ | 0.0013 | 0.0916^b^ | 0.0116 |
| *κ*C 0.5wt% | 0.8514^a^ | 0.0139 | 0.0486^c^ | 0.0003 | 0.0294^bc^ | 0.0011 |
| *κ*C 1.0wt% | 0.5053^b^ | 0.1041 | 0.0107^c^ | 0.0001 | 0.0128^bc^ | 0.0232 |
| **(b)** | **Friction coefficient of CS1** | | | | | |
|  | Boundary lubrication regime | | Mixed lubrication regime | | Mixed lubrication regime | |
|  | (0.005 m s^-1^) | | (0.05 m s^-1^) | | (0.1 m s^-1^) | |
|  | Mean | SD | Mean | SD | Mean | SD |
| CS1 0.5 wt% | 0.6486^a^ | 0.1163 | 0.8137^a^ | 0.0638 | 0.5009^a^ | 0.0662 |
| CS1 1.0 wt% | 0.6907^a^ | 0.0113 | 0.7859^a^ | 0.1906 | 0.5552^ab^ | 0.0617 |
| CS1 2.0 wt% | 0.4542^a^ | 0.1291 | 0.6717^a^ | 0.0307 | 0.3984^ac^ | 0.0323 |
| CS1 3.0 wt% | 0.2015^b^ | 0.0554 | 0.6221^a^ | 0.0306 | 0.3637^ac^ | 0.0186 |
| CS1 5.0 wt% | 0.1169^b^ | 0.0099 | 0.1080^b^ | 0.0345 | 0.0810^d^ | 0.0394 |
| **(c)** | **Friction coefficient of 1.65 wt% *κ*C +CS1 mixtures with pure *κ*C or CS1** | | | | | |
|  | Boundary lubrication regime | | Mixed lubrication regime | | Mixed lubrication regime | |
|  | (0.005 m s^-1^) | | (0.05 m s^-1^) | | (0.1 m s^-1^) | |
|  | Mean | SD | Mean | SD | Mean | SD |
| CS1 1.5 wt% + *κ*C 0.15 wt% | 0.9110^ab^ | 0.0086 | 0.1598^b^ | 0.0424 | 0.0351^b^ | 0.0056 |
| CS1 1.5 wt% | 0.6907^a^ | 0.0113 | 0.7859^a^ | 0.1906 | 0.5552^a^ | 0.0617 |
| *κ*C 0.15 wt% | 1.0665^b^ | 0.2000 | 0.3826^b^ | 0.0013 | 0.0916^b^ | 0.0116 |
| **(d)** | **Friction coefficient of 2.75 wt% *κ*C +CS2 or 2.75 wt% *κ*C +CS1 mixtures with pure *κ*C or CS1 or CS2** | | | | | |
|  | Boundary lubrication regime | | Mixed lubrication regime | | Mixed lubrication regime | |
|  | (0.005 m s^-1^) | | (0.05 m s^-1^) | | (0.1 m s^-1^) | |
|  | Mean | SD | Mean | SD | Mean | SD |
| CS1 2.5 wt% + *κ*C 0.25 wt% | 0.4973^a^ | 0.1714 | 0.0444^a^ | 0.0022 | 0.0149^a^ | 0.0011 |
| CS2 2.5 wt% + *κ*C 0.25 wt% | 0.4389^a^ | 0.0186 | 0.1405^a^ | 0.0055 | 0.0664^a^ | 0.0093 |
| CS1 2.5 wt% | 0.4542^a^ | 0.1291 | 0.6717^b^ | 0.0307 | 0.3984^b^ | 0.0323 |
| CS2 2.5 wt% | 0.6907^ab^ | 0.0104 | 0.7859^b^ | 0.1361 | 0.5707^c^ | 0.0270 |
| *κ*C 0.25 wt% | 0.9589^b^ | 0.1036 | 0.2156^a^ | 0.0007 | 0.0522 ^a^ | 0.0061 |

Supplementary Table S2. Mean and standard deviation (SD) of the apparent viscosity at 50 s^-1^ shear rate of *κ*C (a), CS1 (b), the *κ*C + CS1 mixtures with respective concentrations of *κ*C and CS1 (c). Different lower case letters in the same column indicate a statistically significant difference (*p < 0.05*).

| **(a)** | **Apparent viscosity of *κ*C** | |
| --- | --- | --- |
|  | Shear rate | |
|  | (50 s^-1^) | |
|  | Mean | SD |
| *κ*C 0.05 wt% | 0.0011^a^ | 0.0000 |
| *κ*C 0.1 wt% | 0.0011^a^ | 0.0000 |
| *κ*C 0.5wt% | 0.0142^a^ | 0.0003 |
| *κ*C 1.0wt% | 0.2466^b^ | 0.0106 |
| **(b)** | **Apparent viscosity of CS1** | |
|  | Shear rate | |
|  | (50 s^-1^) | |
|  | Mean | SD |
| CS1 0.5 wt% | 0.0008^a^ | 0.0000 |
| CS1 1.0 wt% | 0.0017^a^ | 0.0000 |
| CS1 2.0 wt% | 0.0025^a^ | 0.0001 |
| CS1 3.0 wt% | 0.0210^b^ | 0.0027 |
| CS1 5.0 wt% | 0.1137^c^ | 0.0112 |
| **(c)** | **Apparent viscosity of 1.65 wt% *κ*C + CS1 mixtures and 2.75 wt% *κ*C + CS1 mixtures with pure *κ*C or CS1** | |
|  | Shear rate | |
|  | (50 s^-1^) | |
|  | Mean | SD |
| CS1 1.5 wt% + *κ*C 0.15 wt% | 0.0244^a^ | 0.0009 |
| CS1 1.5 wt% | 0.0021^b^ | 0.0001 |
| *κ*C 0.15 wt% | 0.0078^c^ | 0.0002 |
| CS1 2.5 wt% + *κ*C 0.25 wt% | 0.0808^d^ | 0.0001 |
| CS1 2.5 wt% | 0.0211^a^ | 0.0027 |
| *κ*C 0.25 wt% | 0.0150^e^ | 0.0023 |
